# Supplementary material for: Non-neutral evolution of H3.3-encoding genes occurs without alterations in protein sequence
Source: Sci Rep. 2019 Jun 11;9:8472. doi: 10.1038/s41598-019-44800-4 (PMC6560044; doi:10.1038/s41598-019-44800-4)
Supplement: Supplementary file 1 — Supplementary Information [file 41598_2019_44800_MOESM1_ESM.pdf]

Supplementary information

Non-neutral evolution of H3.3-encoding genes occurs without alterations in protein sequence

Brejnev M. Muhire[1], Matthew A. Booker[1,2] and Michael Y. Tolstorukov[1,2,*]

[1]Department of Molecular Biology, Massachusetts General Hospital and Harvard Medical School, Boston, MA 02114

[2]current address: Dana-Farber Cancer Institute, Boston, MA 02215

*corresponding author: tolstorukov@molbio.mgh.harvard.edu

Supplementary figures

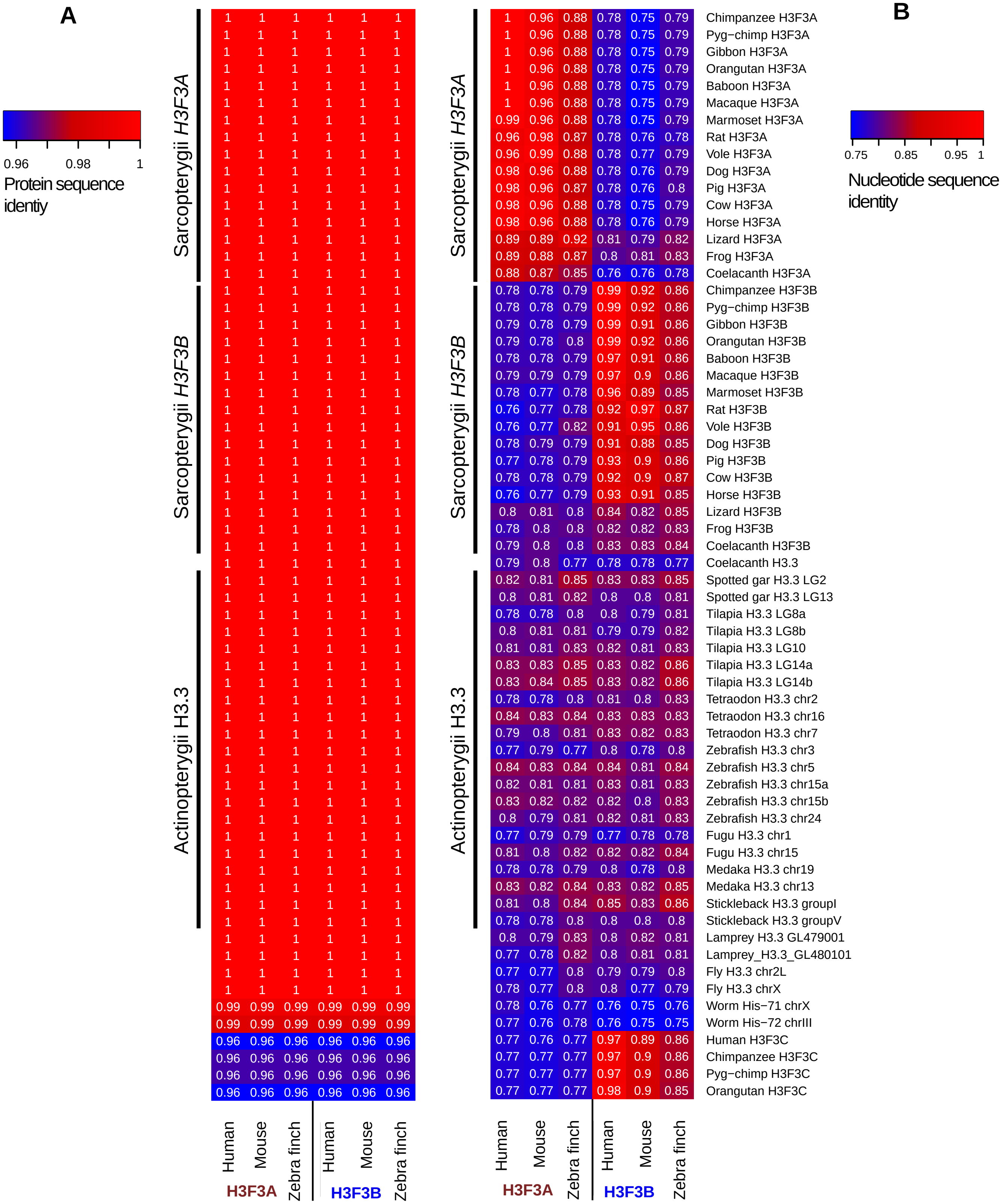

Figure S1. Sequence similarity of H3F3A, H3F3B and H3F3C in metazoa

A. Protein sequence identity computed between human, mouse and zebra finch H3.3 genes (H3F3A and H3F3B) and the H3.3 genes in analyzed metazoa genomes. The hominid-specific H3F3C is included at the bottom.

B. Similar identities as in (A) computed using coding DNA sequences.

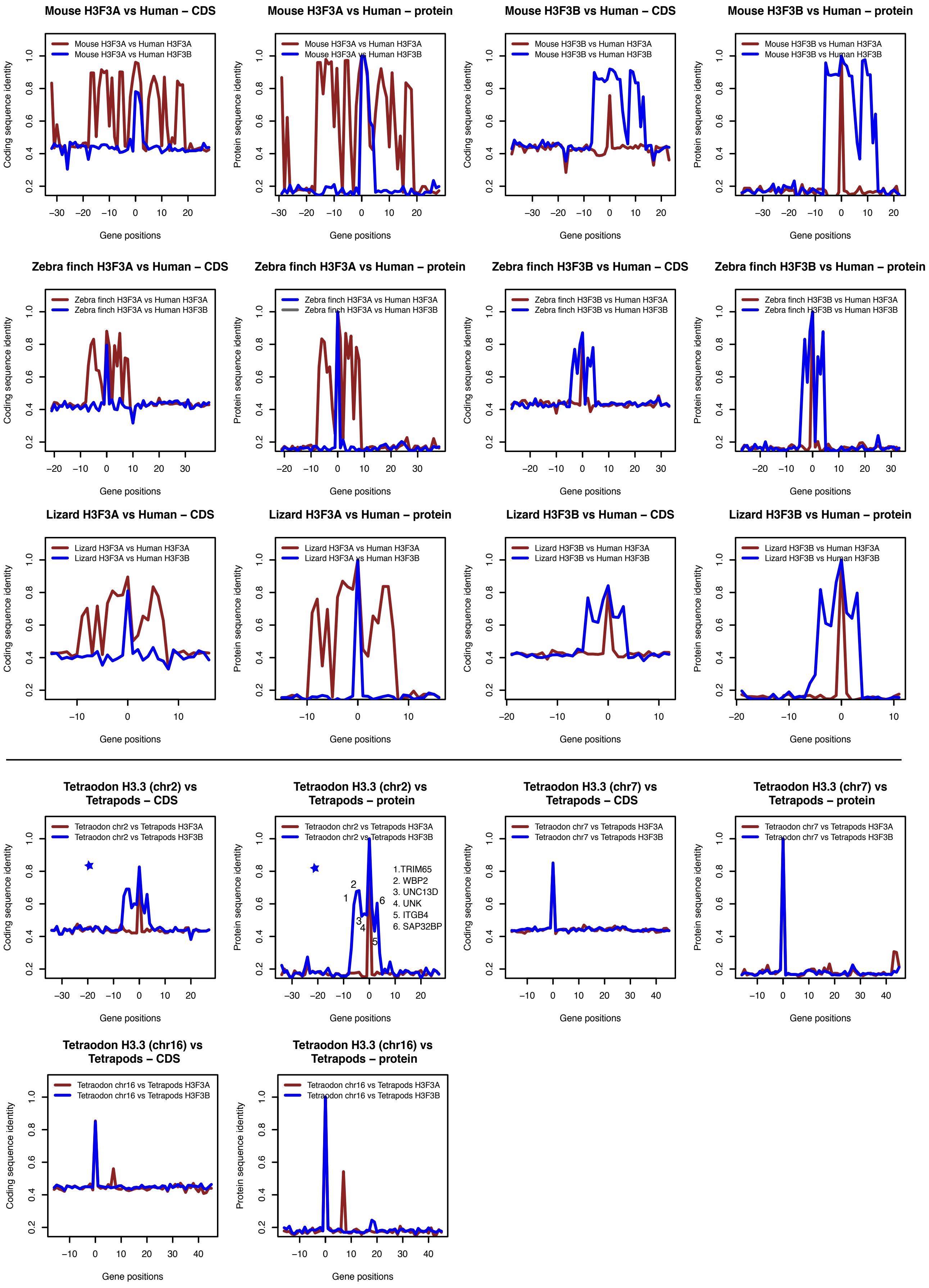
Mouse H3F3A vs Human – CDS
Mouse H3F3A vs Human H3F3A
Mouse H3F3A vs Human H3F3B
Coding sequence identity
Gene positions
Mouse H3F3A vs Human – protein
Mouse H3F3A vs Human H3F3A
Mouse H3F3A vs Human H3F3B
Protein sequence identity
Gene positions
Mouse H3F3B vs Human – CDS
Mouse H3F3B vs Human H3F3A
Mouse H3F3B vs Human H3F3B
Coding sequence identity
Gene positions
Mouse H3F3B vs Human – protein
Mouse H3F3B vs Human H3F3A
Mouse H3F3B vs Human H3F3B
Protein sequence identity
Gene positions
Zebra finch H3F3A vs Human – CDS
Zebra finch H3F3A vs Human H3F3A
Zebra finch H3F3A vs Human H3F3B
Coding sequence identity
Gene positions
Zebra finch H3F3A vs Human – protein
Zebra finch H3F3A vs Human H3F3A
Zebra finch H3F3A vs Human H3F3B
Protein sequence identity
Gene positions
Zebra finch H3F3B vs Human – CDS
Zebra finch H3F3B vs Human H3F3A
Zebra finch H3F3B vs Human H3F3B
Coding sequence identity
Gene positions
Zebra finch H3F3B vs Human – protein
Zebra finch H3F3B vs Human H3F3A
Zebra finch H3F3B vs Human H3F3B
Protein sequence identity
Gene positions
Lizard H3F3A vs Human – CDS
Lizard H3F3A vs Human H3F3A
Lizard H3F3A vs Human H3F3B
Coding sequence identity
Gene positions
Lizard H3F3A vs Human – protein
Lizard H3F3A vs Human H3F3A
Lizard H3F3A vs Human H3F3B
Protein sequence identity
Gene positions
Lizard H3F3B vs Human – CDS
Lizard H3F3B vs Human H3F3A
Lizard H3F3B vs Human H3F3B
Coding sequence identity
Gene positions
Lizard H3F3B vs Human – protein
Lizard H3F3B vs Human H3F3A
Lizard H3F3B vs Human H3F3B
Protein sequence identity
Gene positions
Tetraodon H3.3 (chr2) vs Tetrapods – CDS
Tetraodon chr2 vs Tetrapods H3F3A
Tetraodon chr2 vs Tetrapods H3F3B
Coding sequence identity
Gene positions
Tetraodon H3.3 (chr2) vs Tetrapods – protein
Tetraodon chr2 vs Tetrapods H3F3A
Tetraodon chr2 vs Tetrapods H3F3B
1.TRIM65
2. WBP2
3. UNC13D
4. UNK
5. ITGB4
6. SAP32BP
Protein sequence identity
Gene positions
Tetraodon H3.3 (chr7) vs Tetrapods – CDS
Tetraodon chr7 vs Tetrapods H3F3A
Tetraodon chr7 vs Tetrapods H3F3B
Coding sequence identity
Gene positions
Tetraodon H3.3 (chr7) vs Tetrapods – protein
Tetraodon chr7 vs Tetrapods H3F3A
Tetraodon chr7 vs Tetrapods H3F3B
Protein sequence identity
Gene positions
Tetraodon H3.3 (chr16) vs Tetrapods – CDS
Tetraodon chr16 vs Tetrapods H3F3A
Tetraodon chr16 vs Tetrapods H3F3B
Coding sequence identity
Gene positions
Tetraodon H3.3 (chr16) vs Tetrapods – protein
Tetraodon chr16 vs Tetrapods H3F3A
Tetraodon chr16 vs Tetrapods H3F3B
Protein sequence identity
Gene positions

Figure S2A

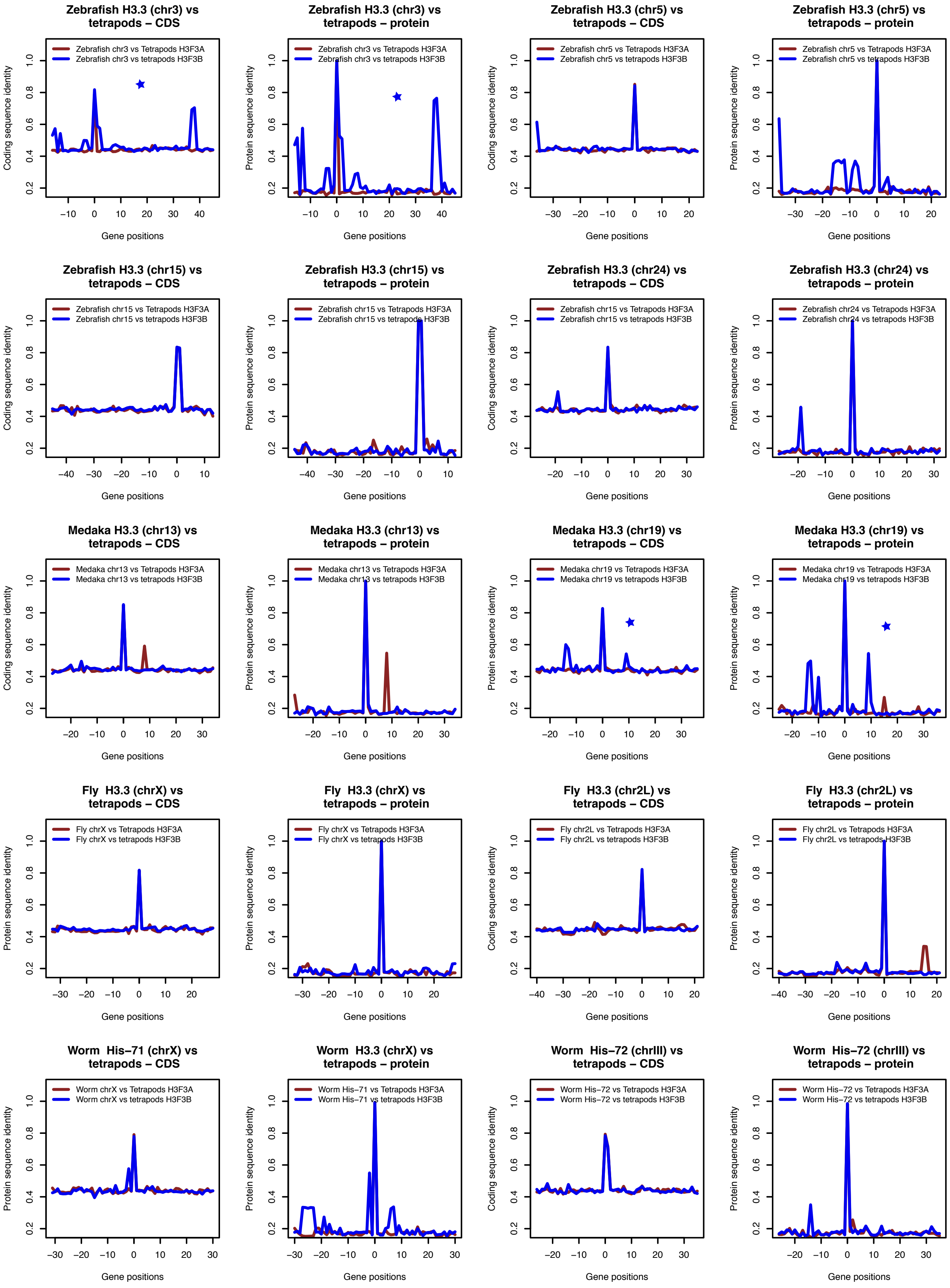

Zebrafish H3.3 (chr3) vs tetrapods – CDS
Zebrafish chr3 vs Tetrapods H3F3A
Zebrafish chr3 vs tetrapods H3F3B
Coding sequence identity
Gene positions
Zebrafish H3.3 (chr3) vs tetrapods – protein
Zebrafish chr3 vs Tetrapods H3F3A
Zebrafish chr3 vs tetrapods H3F3B
Protein sequence identity
Gene positions
Zebrafish H3.3 (chr5) vs tetrapods – CDS
Zebrafish chr5 vs Tetrapods H3F3A
Zebrafish chr5 vs tetrapods H3F3B
Coding sequence identity
Gene positions
Zebrafish H3.3 (chr5) vs tetrapods – protein
Zebrafish chr5 vs Tetrapods H3F3A
Zebrafish chr5 vs tetrapods H3F3B
Protein sequence identity
Gene positions
Zebrafish H3.3 (chr15) vs tetrapods – CDS
Zebrafish chr15 vs Tetrapods H3F3A
Zebrafish chr15 vs tetrapods H3F3B
Coding sequence identity
Gene positions
Zebrafish H3.3 (chr15) vs tetrapods – protein
Zebrafish chr15 vs Tetrapods H3F3A
Zebrafish chr15 vs tetrapods H3F3B
Protein sequence identity
Gene positions
Zebrafish H3.3 (chr24) vs tetrapods – CDS
Zebrafish chr15 vs Tetrapods H3F3A
Zebrafish chr15 vs tetrapods H3F3B
Coding sequence identity
Gene positions
Zebrafish H3.3 (chr24) vs tetrapods – protein
Zebrafish chr24 vs Tetrapods H3F3A
Zebrafish chr24 vs tetrapods H3F3B
Protein sequence identity
Gene positions
Medaka H3.3 (chr13) vs tetrapods – CDS
Medaka chr13 vs Tetrapods H3F3A
Medaka chr13 vs tetrapods H3F3B
Coding sequence identity
Gene positions
Medaka H3.3 (chr13) vs tetrapods – protein
Medaka chr13 vs Tetrapods H3F3A
Medaka chr13 vs tetrapods H3F3B
Protein sequence identity
Gene positions
Medaka H3.3 (chr19) vs tetrapods – CDS
Medaka chr19 vs Tetrapods H3F3A
Medaka chr19 vs tetrapods H3F3B
Protein sequence identity
Gene positions
Medaka H3.3 (chr19) vs tetrapods – protein
Medaka chr19 vs Tetrapods H3F3A
Medaka chr19 vs tetrapods H3F3B
Protein sequence identity
Gene positions
Fly H3.3 (chrX) vs tetrapods – CDS
Fly chrX vs Tetrapods H3F3A
Fly chrX vs tetrapods H3F3B
Protein sequence identity
Gene positions
Fly H3.3 (chrX) vs tetrapods – protein
Fly chrX vs Tetrapods H3F3A
Fly chrX vs tetrapods H3F3B
Protein sequence identity
Gene positions
Fly H3.3 (chr2L) vs tetrapods – CDS
Fly chr2L vs Tetrapods H3F3A
Fly chr2L vs tetrapods H3F3B
Coding sequence identity
Gene positions
Fly H3.3 (chr2L) vs tetrapods – protein
Fly chr2L vs Tetrapods H3F3A
Fly chr2L vs tetrapods H3F3B
Protein sequence identity
Gene positions
Worm His–71 (chrX) vs tetrapods – CDS
Worm chrX vs Tetrapods H3F3A
Worm chrX vs tetrapods H3F3B
Protein sequence identity
Gene positions
Worm H3.3 (chrX) vs tetrapods – protein
Worm His–71 vs Tetrapods H3F3A
Worm His–71 vs tetrapods H3F3B
Protein sequence identity
Gene positions
Worm His–72 (chrIII) vs tetrapods – CDS
Worm His–72 vs Tetrapods H3F3A
Worm His–72 vs tetrapods H3F3B
Coding sequence identity
Gene positions
Worm His–72 (chrIII) vs tetrapods – protein
Worm His–72 vs Tetrapods H3F3A
Worm His–72 vs tetrapods H3F3B
Protein sequence identity
Gene positions

Figure S2B

Figure S2. Synteny around H3F3A and H3F3B genes

A, B Conservation of genes around H3.3 genes between human and other tetrapods (mouse, zebra finch and lizard; top three rows in A) and conservation genes around H3.3 genes between tetrapods and distant organisms (actinopterygian, fly and worm; the last two rows in A and all rows in B). Sequence conservation (identities) was measured for both nucleotide and protein sequences. The brown graph shows identities between genes around tetrapods H3.3 and human *H3F3A*, or genes conserved around non-tetrapod H3.3 and tetrapod *H3F3A* genes. Similarly, the blue graph shows identities between genes conserved around H3.3-encoding gene in a given organism and genes around Human *H3F3B* or tetrapod *H3F3B*. A blue star indicates non-tetrapod gene sharing syntenic genes with tetrapod *H3F3B*. The highest peaks at gene position 0 (100% protein identity for brown and blue lines) represent the identity between a given H3.3 gene and tetrapod *H3F3A* and *H3F3B*.

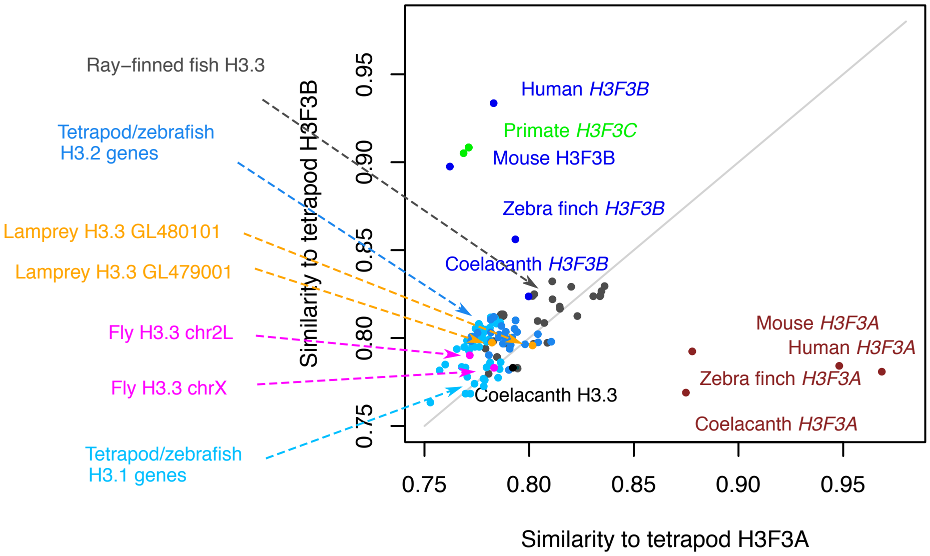

Figure S3. Comparison of tetrapod H3.3 genes to related genes in sarcopterygian and non-sarcopterygian lineages.

Average sequences similarity was estimated for the CDS of tetrapod (human, mouse, zebra finch) H3.3 genes (H3F3A, x-axis and H3F3B, y-axis) and CDS of each H3.3 gene in sarcopterygians, actinopterygians and more distant organisms (lamprey, fly). Additionally, CDS of tetrapod and zebrafish H3.1 and H3.2 genes were included in this analysis. Each point represents a gene and the organism name is written in the matching color. The sequence similarity represents percentage of the identical nucleotides in the sequence.

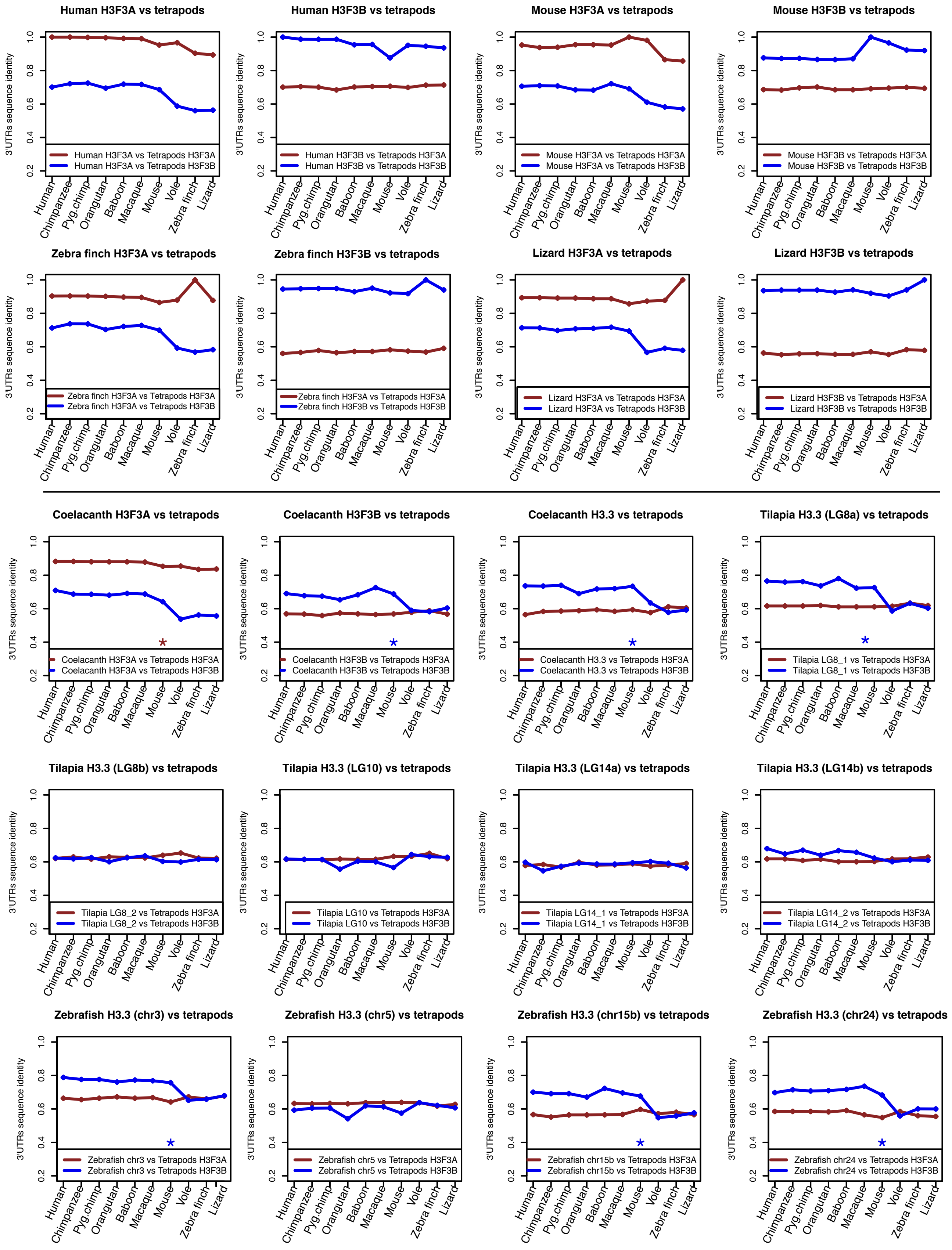
3'UTRs sequence identity
0.2
0.4
0.6
0.8
1.0
Human
Chimpanzee
Pyg.chimp
Orangutan
Baboon
Macaque
Mouse
Vole
Zebra finch
Lizard
Human H3F3A vs tetrapods
Human H3F3A vs Tetrapods H3F3A
Human H3F3A vs Tetrapods H3F3B
Human H3F3B vs tetrapods
Human H3F3B vs Tetrapods H3F3A
Human H3F3B vs Tetrapods H3F3B
Mouse H3F3A vs tetrapods
Mouse H3F3A vs Tetrapods H3F3A
Mouse H3F3A vs Tetrapods H3F3B
Mouse H3F3B vs tetrapods
Mouse H3F3B vs Tetrapods H3F3A
Mouse H3F3B vs Tetrapods H3F3B
Zebra finch H3F3A vs tetrapods
Zebra finch H3F3A vs Tetrapods H3F3A
Zebra finch H3F3A vs Tetrapods H3F3B
Zebra finch H3F3B vs tetrapods
Zebra finch H3F3A vs Tetrapods H3F3A
Zebra finch H3F3B vs Tetrapods H3F3B
Lizard H3F3A vs tetrapods
Lizard H3F3A vs Tetrapods H3F3A
Lizard H3F3A vs Tetrapods H3F3B
Lizard H3F3B vs tetrapods
Lizard H3F3B vs Tetrapods H3F3A
Lizard H3F3B vs Tetrapods H3F3B
Coelacanth H3F3A vs tetrapods
Coelacanth H3F3A vs Tetrapods H3F3A
Coelacanth H3F3A vs Tetrapods H3F3B
Coelacanth H3F3B vs tetrapods
Coelacanth H3F3B vs Tetrapods H3F3A
Coelacanth H3F3B vs Tetrapods H3F3B
Coelacanth H3.3 vs tetrapods
Coelacanth H3.3 vs Tetrapods H3F3A
Coelacanth H3.3 vs Tetrapods H3F3B
Tilapia H3.3 (LG8a) vs tetrapods
Tilapia LG8_1 vs Tetrapods H3F3A
Tilapia LG8_1 vs Tetrapods H3F3B
Tilapia H3.3 (LG8b) vs tetrapods
Tilapia LG8_2 vs Tetrapods H3F3A
Tilapia LG8_2 vs Tetrapods H3F3B
Tilapia H3.3 (LG10) vs tetrapods
Tilapia LG10 vs Tetrapods H3F3A
Tilapia LG10 vs Tetrapods H3F3B
Tilapia H3.3 (LG14a) vs tetrapods
Tilapia LG14_1 vs Tetrapods H3F3A
Tilapia LG14_1 vs Tetrapods H3F3B
Tilapia H3.3 (LG14b) vs tetrapods
Tilapia LG14_2 vs Tetrapods H3F3A
Tilapia LG14_2 vs Tetrapods H3F3B
Zebrafish H3.3 (chr3) vs tetrapods
Zebrafish chr3 vs Tetrapods H3F3A
Zebrafish chr3 vs Tetrapods H3F3B
Zebrafish H3.3 (chr5) vs tetrapods
Zebrafish chr5 vs Tetrapods H3F3A
Zebrafish chr5 vs Tetrapods H3F3B
Zebrafish H3.3 (chr15b) vs tetrapods
Zebrafish chr15b vs Tetrapods H3F3A
Zebrafish chr15b vs Tetrapods H3F3B
Zebrafish H3.3 (chr24) vs tetrapods
Zebrafish chr24 vs Tetrapods H3F3A
Zebrafish chr24 vs Tetrapods H3F3B

Figure S4A

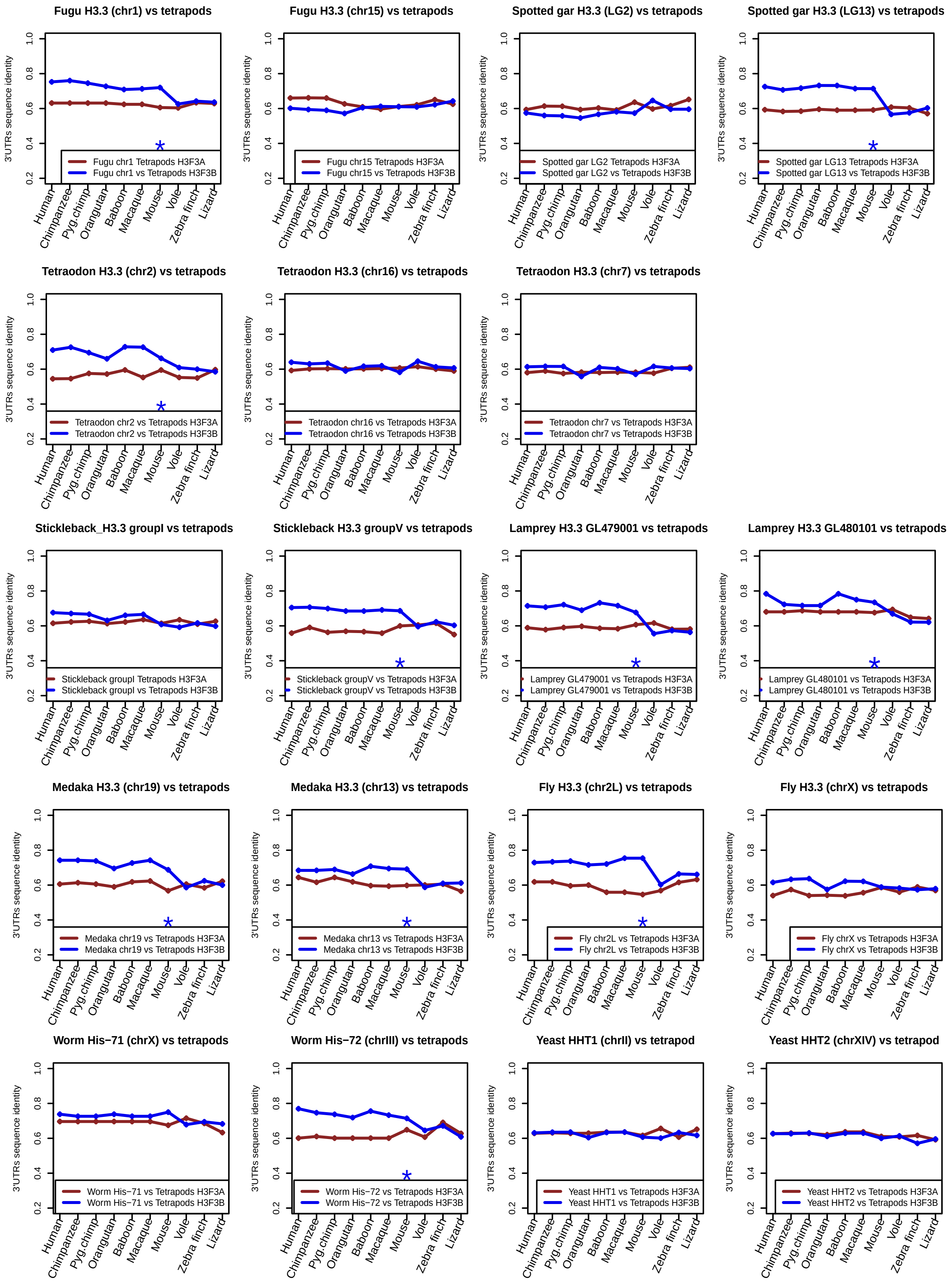
Fugu H3.3 (chr1) vs tetrapods
Fugu chr1 Tetrapods H3F3A
Fugu chr1 vs Tetrapods H3F3B
Fugu H3.3 (chr15) vs tetrapods
Fugu chr15 Tetrapods H3F3A
Fugu chr15 vs Tetrapods H3F3B
Spotted gar H3.3 (LG2) vs tetrapods
Spotted gar LG2 Tetrapods H3F3A
Spotted gar LG2 vs Tetrapods H3F3B
Spotted gar H3.3 (LG13) vs tetrapods
Spotted gar LG13 Tetrapods H3F3A
Spotted gar LG13 vs Tetrapods H3F3B
Tetraodon H3.3 (chr2) vs tetrapods
Tetraodon chr2 vs Tetrapods H3F3A
Tetraodon chr2 vs Tetrapods H3F3B
Tetraodon H3.3 (chr16) vs tetrapods
Tetraodon chr16 vs Tetrapods H3F3A
Tetraodon chr16 vs Tetrapods H3F3B
Tetraodon H3.3 (chr7) vs tetrapods
Tetraodon chr7 vs Tetrapods H3F3A
Tetraodon chr7 vs Tetrapods H3F3B
Stickleback_H3.3 groupI vs tetrapods
Stickleback groupI Tetrapods H3F3A
Stickleback groupI vs Tetrapods H3F3B
Stickleback H3.3 groupV vs tetrapods
Stickleback groupV Tetrapods H3F3A
Stickleback groupV vs Tetrapods H3F3B
Lamprey H3.3 GL479001 vs tetrapods
Lamprey GL479001 vs Tetrapods H3F3A
Lamprey GL479001 vs Tetrapods H3F3B
Lamprey H3.3 GL480101 vs tetrapods
Lamprey GL480101 vs Tetrapods H3F3A
Lamprey GL480101 vs Tetrapods H3F3B
Medaka H3.3 (chr19) vs tetrapods
Medaka chr19 vs Tetrapods H3F3A
Medaka chr19 vs Tetrapods H3F3B
Medaka H3.3 (chr13) vs tetrapods
Medaka chr13 vs Tetrapods H3F3A
Medaka chr13 vs Tetrapods H3F3B
Fly H3.3 (chr2L) vs tetrapods
Fly chr2L vs Tetrapods H3F3A
Fly chr2L vs Tetrapods H3F3B
Fly H3.3 (chrX) vs tetrapods
Fly chrX vs Tetrapods H3F3A
Fly chrX vs Tetrapods H3F3B
Worm His−71 (chrX) vs tetrapods
Worm His−71 vs Tetrapods H3F3A
Worm His−71 vs Tetrapods H3F3B
Worm His−72 (chrIII) vs tetrapods
Worm His−72 vs Tetrapods H3F3A
Worm His−72 vs Tetrapods H3F3B
Yeast HHT1 (chrII) vs tetrapod
Yeast HHT1 vs Tetrapods H3F3A
Yeast HHT1 vs Tetrapods H3F3B
Yeast HHT2 (chrXIV) vs tetrapod
Yeast HHT2 vs Tetrapods H3F3A
Yeast HHT2 vs Tetrapods H3F3B
3'UTRs sequence identity
0.2
0.4
0.6
0.8
1.0
Human
Chimpanzee
Pyg.chimp
Orangutan
Baboon
Macaque
Mouse
Vole
Zebra finch
Lizard

Figure S4B

Figure S4. Conservation of H3.3 genes 3'UTRs in metazoa

A, B H3.3 gene 3'UTRs sequence conservation among tetrapod organisms (top two rows in A) and H3.3 gene 3'UTRs conservation between tetrapods and non-tetrapod organisms (coelacanth, actinopterygians, fly and worm; last three rows in A and all rows in B). The blue line represents the sequence identity between a given H3.3 gene's UTR and the *H3F3B* 3'UTR from ten tetrapod organisms (x-axis). Similarly, the brown line shows the sequence identity between the 3' UTR of a given gene and the 3'UTR of tetrapod *H3F3A* genes. A blue asterisk indicates non-tetrapod H3.3 genes for which UTRs sequence is more similar to that of tetrapod *H3F3B* (blue) or tetrapod *H3F3A* (brown) in the majority tetrapod organisms included.

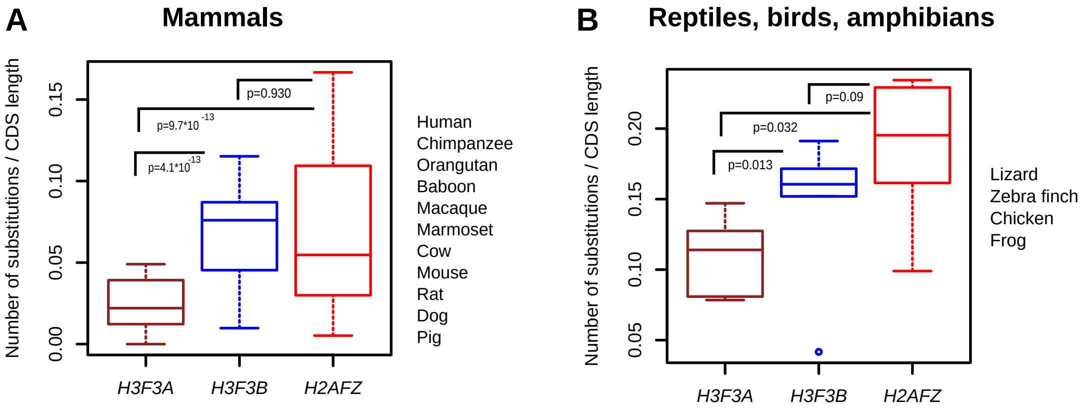

Figure S5. CDS conservation of histone variant genes in tetrapods

A, B. Pairwise nucleotide substitution scores (genetic distances) computed for two H3.3 genes (H3F3A, brown and H3F3B, blue), and H2AFZ gene (red) which was included in this analysis for comparison. The analysis was performed separately for mammalian (A) and other tetrapod genomes (reptiles, birds and amphibians; B). Comparison of scores was done with a Wilcox sum rank test and the organisms included are shown on the right side of each figure.

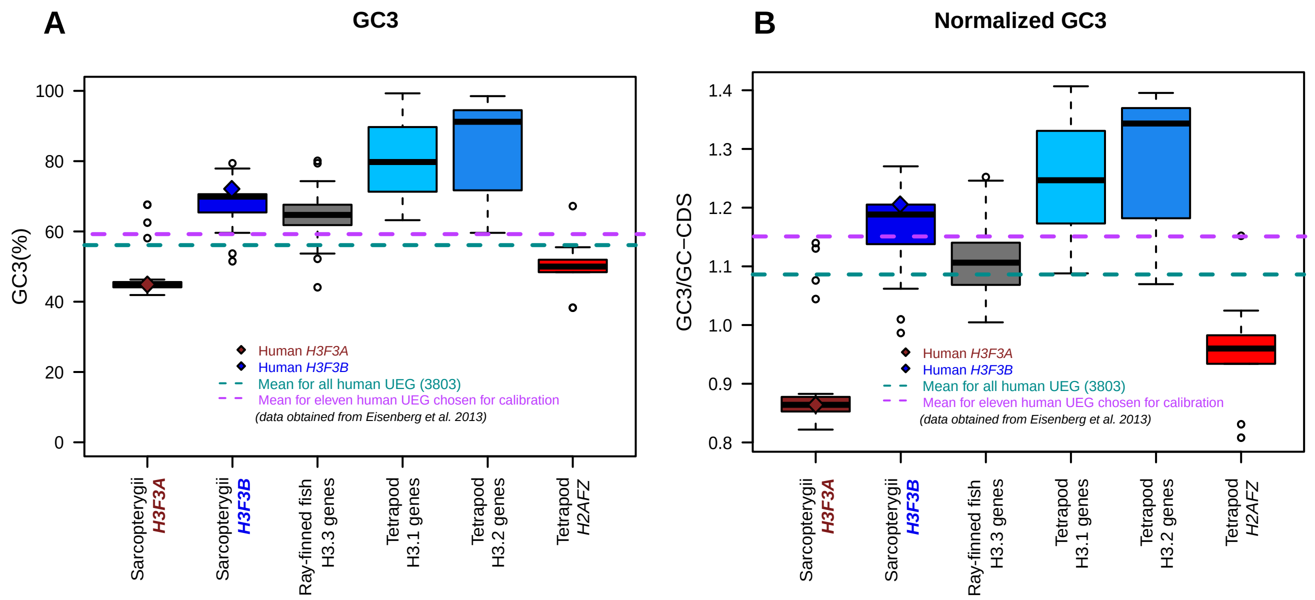

Figure S6. GC3 of H3.3 CDS.

A. Distribution of GC3 scores for tetrapod H3F3A and H3F3B, actinopterygian H3.3, tetrapod/zebrafish H3.1, H3.2 and H2AFZ genes. **B.** Distribution of normalized GC3 scores. For a particular gene, the normalized GC3 scores is the GC-content at codon 3rd position (GC3) divided by the GC-content of the whole gene (GC-CDS). The dashed lines represents average correlation computed for human ubiquitously expressed genes (UEG)[46]. Cyan: the full set of UEGs; Magenta: a subset of eleven UEGs proposed because of their highly uniform and strong expression across human cell types.

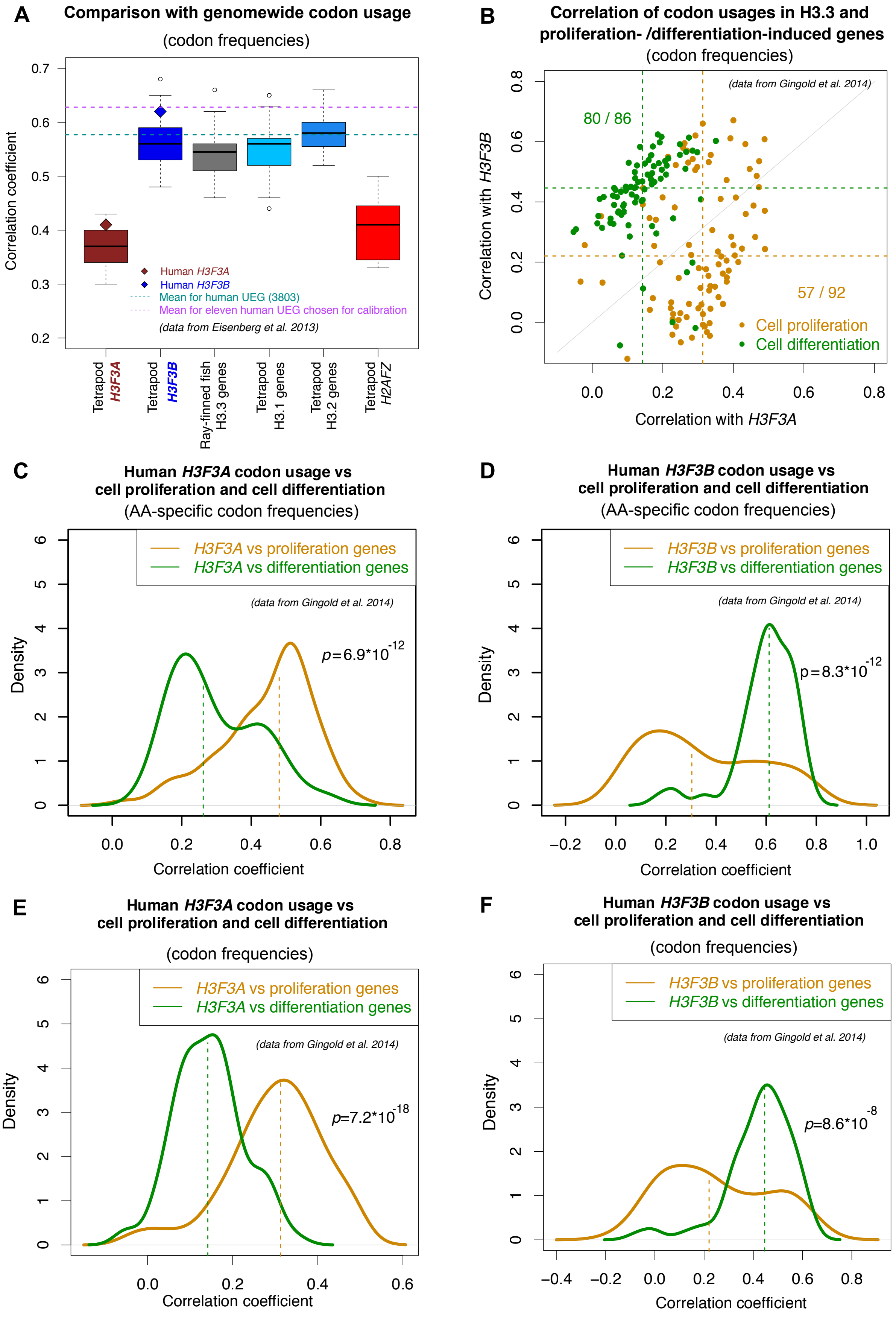
A
Comparison with genomewide codon usage
(codon frequencies)
Correlation coefficient
0.7
0.6
0.5
0.4
0.3
0.2
Human H3F3A
Human H3F3B
Mean for human UEG (3803)
Mean for eleven human UEG chosen for calibration
(data from Eisenberg et al. 2013)
Tetrapod H3F3A
Tetrapod H3F3B
Ray-finned fish H3.3 genes
Tetrapod H3.1 genes
Tetrapod H3.2 genes
Tetrapod H2AFZ
B
Correlation of codon usages in H3.3 and
proliferation- /differentiation-induced genes
(codon frequencies)
(data from Gingold et al. 2014)
80 / 86
57 / 92
Cell proliferation
Cell differentiation
Correlation with H3F3B
Correlation with H3F3A
0.0
0.2
0.4
0.6
0.8
C
Human H3F3A codon usage vs
cell proliferation and cell differentiation
(AA-specific codon frequencies)
H3F3A vs proliferation genes
H3F3A vs differentiation genes
(data from Gingold et al. 2014)
p=6.9*10^-12
Density
Correlation coefficient
D
Human H3F3B codon usage vs
cell proliferation and cell differentiation
(AA-specific codon frequencies)
H3F3B vs proliferation genes
H3F3B vs differentiation genes
(data from Gingold et al. 2014)
p=8.3*10^-12
Density
Correlation coefficient
E
Human H3F3A codon usage vs
cell proliferation and cell differentiation
(codon frequencies)
H3F3A vs proliferation genes
H3F3A vs differentiation genes
(data from Gingold et al. 2014)
p=7.2*10^-18
Density
Correlation coefficient
F
Human H3F3B codon usage vs
cell proliferation and cell differentiation
(codon frequencies)
H3F3B vs proliferation genes
H3F3B vs differentiation genes
(data from Gingold et al. 2014)
p=8.6*10^-8
Density
Correlation coefficient

Figure S7

Figure S7. Distinct codon usage preferences in the H3.3 genes

A. Correlation between codon usage in the genes specified at x-axis and the genome-wide codon usage. The box plots represent the lineage distributions of the correlation coefficients calculated for the 'codon frequencies' of a corresponding gene with those estimated genome-wide (e.g. all tetrapod H3F3A genes vs. genome-wide frequencies). The brown and blue diamonds provide reference for human *H3F3A* and *H3F3B* respectively. The dashed lines represent average correlation computed for human ubiquitously expressed genes (UEG)[46]. Cyan: the full set of UEGs; Magenta: a subset of eleven UEGs proposed because of their highly uniform and strong expression across human cell types. **B.** Correlation of human H3F3A and H3F3B 'codon frequencies' with those computed for the genes associated with cell proliferation (orange) and cell differentiation (green)[43]. Each dot represents an individual gene from the corresponding group. The dotted lines indicate the correlation coefficient medians for each group and the H3.3 gene. **C,D.** Comparison of distributions of correlation scores of human H3F3A 'amino-acid specific codon frequencies' and those computed for genes associated with cell proliferation (orange), and cell differentiation (green)[43] (C). Similar comparisons performed for Human H3F3B and genes associated with cell proliferation (orange) and differentiation (green) (D). A vertical dashed line represents median of correlation scores obtained for a given H3.3 gene and a given group of genes. Comparison of scores was done with a Mann-Whitney test. **E,F.** Similar comparisons as in C and D respectively, performed based on 'codon frequencies'.

Supplementary table

Table S1. Number of H3.3 encoding genes per organism

No.	Name	Scientific name	Class / phylum	No. of H3.3
1	Human	*Homo sapiens*	Sarcopterygii	2
2	Chimpanzee	*Pan troglodytes*	Sarcopterygii	2
3	Pygmy chimpanzee	*Pan paniscus*	Sarcopterygii	2
4	Gorilla	*Gorilla gorilla*	Sarcopterygii	2
5	Orangutan	*Pongo abelii*	Sarcopterygii	2
6	Gibbon	*Nomascus leucogenys*	Sarcopterygii	2
7	Baboon	*Papio anubis*	Sarcopterygii	2
8	Macaque	*Macaca mulatta*	Sarcopterygii	2
9	Marmoset	*Callithrix jacchus*	Sarcopterygii	2
10	Mouse	*Mus musculus*	Sarcopterygii	2
11	Rat	*Rattus norvegicus*	Sarcopterygii	2
12	Prairie Vole	*Microtus ochrogaster*	Sarcopterygii	2
13	Opossum	*Monodelphis domestica*	Sarcopterygii	2
14	Dog	*Canis familialis*	Sarcopterygii	2
15	Pig	*Sus scrofa*	Sarcopterygii	2
16	Cow	*Bos taurus*	Sarcopterygii	2
17	Horse	*Equus caballus*	Sarcopterygii	2
18	Chicken	*Gallus gallus*	Sarcopterygii	2
19	Zebra Finch	*Taeniopygia guttata*	Sarcopterygii	2
20	Lizard	*Anolis carolinensis*	Sarcopterygii	2
21	Frog	*Xenopus tropicalis*	Sarcopterygii	2
22	Coelacanth	*Latimeria chalumnae*	Sarcopterygii	3
23	Tilapia	*Oreochromis niloticus*	Actinopterygii	5
24	Zebrafish	*Danio rerio*	Actinopterygii	5
25	Fugu	*Takifugu rubripes*	Actinopterygii	3
26	Medaka	*Oryzias latipes*	Actinopterygii	3
27	Tetraodon	*Tetraodon nigroviridis*	Actinopterygii	3
28	Spotted gar	*Lepisosteus oculatus*	Actinopterygii	3
29	Stickleback	*Gasterosteus aculeatus*	Actinopterygii	3
30	Lamprey	*Petromyzon marinus*	Hyperoartia	2
31	Fruit fly	*Drosophila melanogaster*	Insecta	2
32	Worm	*Caenorhabditis elegans*	Nematoda	2
